# Supplementary material for: Collaborative intelligence in AI: Evaluating the performance of a council of AIs on the USMLE
Source: PLOS Digit Health. 2025 Oct 9;4(10):e0000787. doi: 10.1371/journal.pdig.0000787 (PMC12510544; doi:10.1371/journal.pdig.0000787)
Supplement: S2 File — (ZIP) [file pdig.0000787.s002.zip › S2 Data Raw data files containing unprocessed question inputs and LLM outputs including transcripts of deliberations/STEP3_Question-105-1_Type-MC-NJ.docx]

**Facilitator** **[Round 1]:** A 67-year-old man with Parkinson disease is admitted to the hospital for treatment of pneumonia. The patient's daughter, who is visiting the patient, says he has had increased lethargy for the past day and decreased ambulation during the past 6 months. She also says that there are times during the day when his tremors increase in severity, although he continues to care for himself at home. Medical history is also remarkable for hypertension. Medications include hydrochlorothiazide, atenolol, levodopa, and carbidopa. He is 168 cm (5 ft 6 in) tall and weighs 78 kg (172 lb); BMI is 28 kg/m². Vital signs are temperature 38.9°C (102.0°F), pulse 60/min supine and 68/min standing, respirations 22/min, and blood pressure 100/60 mm Hg supine and 80/50 mm Hg standing. The patient appears ill and older than his stated age. He is fully oriented but lethargic. Auscultation of the chest discloses rhonchi in the right mid lung field. Abdominal examination discloses no abnormalities. Neurologic examination discloses masked facies, bradykinesia, and cogwheel rigidity; gait was not assessed on admission. Chest x-ray shows a right lower lobe infiltrate. ECG shows no abnormalities. Appropriate intravenous antibiotic therapy is initiated. Prior to discharge, which of the following is the most appropriate step? (A) Obtain CT scan of the chest (B) Obtain a swallowing evaluation (C) Place a percutaneous endoscopic gastrostomy (PEG) tube (D) Prescribe fludrocortisone (E) Prescribe prophylactic levofloxacin

**AI_E:** Restating the question: We're asked to determine the most appropriate step before discharging a 67-year-old Parkinsonian patient who was admitted for pneumonia treatment. The patient had increased lethargy and decreased mobility over the past 6 months, occasional severe tremors, yet remained independent. The patient also had hypertension, was on multiple medications including hydrochlorothiazide, atenolol, levodopa, and carbidopa, and had an infection localized in the right mid lung field. Planning: The assumptions here are mainly around the critical health management choices we need to undertake before discharging the patient. Some decisions revolve around further diagnostic actions ((A) obtaining a CT scan of the chest or (B) obtaining a swallowing evaluation), others consider the patient's feeding management (C) placing a PEG tube, while others focus on prophylactic and treatment measures ((D) prescribing fludrocortisone or (E) prescribing prophylactic levofloxacin). Clinical Reasoning: Parkinson's disease is a neurodegenerative disorder that predominantly affects the motor system. The increase in the patient's tremors and reduced ambulation can be attributed to its progressive nature. Another noteworthy aspect is the occurrence of swallow difficulties (dysphagia) in Parkinson's, raising risk for aspiration pneumonia - which could explain our patient's condition. The antibiotics treatment for pneumonia is insufficient to address this underlying issue. Concluding: Given his Parkinson's disease, the patient's swallowing mechanism may be compromised, predisposing him to recurrent aspiration and subsequently, pneumonia. The most clinically sound decision would be to address this potential source of recurrent pneumonia. Thus, the response is: (B) Obtain a swallowing evaluation. Clinical swallowing evaluations will help assess the safety and efficiency of a patient's swallow and determine the need for further assessment or intervention.

**AI_B:** RESTATING THE QUESTION: We are looking to determine the best next course of action for a 67-year-old male Parkinson's patient who has been admitted for pneumonia treatment. The man has a history of sudden onset lethargy, decreased ambulation, hypertension, and progressive Parkinson's symptoms like severe tremors. He is being treated with respective medications. PLANNING: Assumptions are that the man's ill state is due to the pneumonia, and his symptoms will improve after treatment. However, Parkinson's Disease affects swallowing process and disease progression combined with increased lethargy may lead to swallowing difficulties increasing the risk of aspiration, and thus recurrent pneumonia. As he maintained activity at home till now, this indicates a more likely swallowing dysfunction over a general physical decline. Hence, evaluating the swallowing capability should be clinically prioritized. CLINICAL REASONING: Parkinson's disease is a neurodegenerative disorder, which along with motor symptoms like tremors, bradykinesia, also commonly presents with dysphagia due to the delayed and uncoordinated swallowing movements. This poses aspiration risks, that is, unintentional entry of food or liquids into the airways, frequently leading to pneumonia - the condition for which the man was admitted. His increased lethargy and inability to nomalize ambulation could indicate this swallowing difficulty rather than being just fatigue. The chances are considerable considering the chronicity and severity of disease, as noted from his extending symptoms, and as he continues to feed himself. CONCLUDING: Considering the Parkinson condition and the associated risks due to dysphagia, it's important to consider the well-being of the patient in a long-term perspective. An evaluation of the patient's swallowing ability can help to identify and subsequently manage potential ingestion or aspiration problems. This can in turn prevent recurrent pneumonia or other pulmonary complications, thus enhancing his quality of life. RESPONSE: The most appropriate step prior to discharge is to (B) Obtain a swallowing evaluation.

**AI_A:** RESTATING THE QUESTION: A man with Parkinson's disease, known for causing movement abnormalities, has been hospitalized for pneumonia. He's lethargic and his ability to move has decreased over the past six months. His daughter reports periods of increased tremors. The pneumonia is being treated, but we need to plan for his discharge. Among the offered choices, we have options for further diagnostic testing (CT scan and swallowing evaluation), a procedural intervention (PEG tube), and medication interventions (fludrocortisone and levofloxacin). The medication options are for addressing low blood pressure and pneumonia respectively. PLANNING: There are several assumptions in this scenario, such as the patient's pneumonia might have resulted from aspiration due to difficulty in swallowing (dysphagia), common in Parkinson's disease. Also, the patient's hypotension is a result of his medications or lengthy hospital stay, and not a symptom of Parkinson's disease. Another assumption is that the prophylactic levofloxacin is to prevent recurrence of pneumonia, which might not be the best strategy given potential overuse of antibiotics. Lastly, the assumption about PEG tube is that he needs a long-term solution for nutrition. CLINICAL REASONING: The hypothesis would be to rule out dysphagia, a common symptom in Parkinson's disease, which could be causing the patient's pneumonia due to aspiration. The consideration for a CT scan of the chest needs considering if there are unresolved or questionable findings on the chest x-ray, which is not the case here. The placement of a PEG tube might be premature without first evaluating swallowing function. Fludrocortisone is often used in neurogenic orthostatic hypotension, a common finding in Parkinson's disease, but this patient's drop in blood pressure could be due to his current illness, medications, or dehydration rather than being a symptom of his disease. Prescribing prophylactic levofloxacin doesn't support prudent antibiotic stewardship and might have adverse effects, especially in the elderly. CONCLUDING: While there are a variety of interventions available for this patient, addressing the most immediate and potentially recurring problem seems the most prudent. Thus, performing a swallowing evaluation would help monitor for dysphagia, which could contribute to future pneumonia events. RESPONSE: Given the factors discussed above, before the patient is discharged, the most appropriate next step would be to perform a swallowing evaluation (B) to assess the possible contributing impacts of dysphagia on his pneumonia, as well as to guide future nutritional needs and strategies.

**AI_C:** RESTATING THE QUESTION: This elderly Parkinson's patient has been hospitalized due to pneumonia. He has also been experiencing an increased state of lethargy and a decline in his mobility over recent months. With notable symptoms such as tremors often worsening throughout the day, these are all hallmark signs of his Parkinson's disease. Despite this, he has managed to maintain self-care at home. He also has a history of high blood pressure, and his medications include hydrochlorothiazide, atenolol, levodopa, and carbidopa. His physical examination results have revealed rhonchi in the right mid lung field, suggestive of his admitted pneumonia diagnosis, as well as signs of his progressive Parkinson's disease. Given his medical situation, we are considering the most appropriate next step in managing his condition before he is discharged from the hospital. PLANNING: One assumption we could make is that his pneumonia needs further investigation via CT scan, hence option (A). Alternatively, the increased lethargy and decline in mobility could be suspect for dysphagia, often associated with Parkinson's disease, and could rationalize a swallowing evaluation for the safety of the patient (option B). Yet another assumption is that this patient’s decreased ambulation might cause malnutrition leading to the option of placing a PEG tube (option C). Considering the drop in blood pressure upon standing, it's also plausible to contemplate prescribing fludrocortisone (option D) or with his pneumonia, prescribing prophylactic levofloxacin could be an option (option E). CLINICAL REASONING: Given the patient's Parkinson's disease, the odds of dysphagia (difficulty swallowing) are high. Dysphagia can cause multiple problems, such as malnutrition, dehydration, and aspiration pneumonia. The latter, aspiration pneumonia, occurs when food, saliva, liquids, or vomit is breathed into the lungs or airways leading to the lungs, instead of being swallowed into the esophagus and stomach. Given our patient's recent pneumonia, it would be prudent to evaluate if aspirational episodes might have contributed to this condition, or could pose a future risk. Therefore, a swallowing evaluation (option B), seems a reasonable next step to ensure patient safety. CONCLUDING: Considering all assumptions, the presence of Parkinson's disease, recent pneumonia, and decreased ambulation, alongside the patient's ability to self-nourish, suggest that dysphagia related to Parkinson's disease could be a significant factor worth investigating. This leads me to prioritize a swallowing evaluation to help prevent future aspiration pneumonia occurrences. RESPONSE: On the basis of the clinical presentation and the highest clinical priorities for patient safety, the most appropriate next step before discharging this Parkinson's patient after treatment for pneumonia should be to (B) obtain a swallowing evaluation.

**AI_D:** RESTATING THE QUESTION: We need to determine which of the listed options is the most appropriate next step for our patient, a 67-year-old male with Parkinson's disease, hypertension and pneumonia, prior to his discharge from the hospital. He is taking hydrochlorothiazide, atenolol, levodopa, and carbidopa for his existing conditions. He displays increased lethargy and decreased ambulation, notably with occasions of increased tremors. In Parkinson's disease, degeneration of dopamine-producing neurons cause symptoms like tremors, bradykinesia, rigidity and postural instability. Levodopa and carbidopa are used as a combined therapy to increase dopamine levels and reduce symptoms. PLANNING: The assumptions here are that all current medical management efforts are adequate and that the pneumonia has been dealt with sufficiently, allowing the patient to be discharged. However, what requires examination in this context is the exacerbating Parkinson's symptoms, particularly the presence of increased tremors and lethargy, which might indicate problems beyond the simple progression of Parkinson's disease. Reduced ambulation is a recognized manifestation of advanced Parkinson's, but when coupled with swallowing difficulties (dysphagia), it could lead to an increased risk of aspiration, which may have resulted in the current episode of pneumonia. Given his medical history and symptoms, we might hypothesize that the most appropriate further investigation would be related to the possible dysphagia. CLINICAL REASONING: The options offered for consideration primarily deal with either further exploration of the respiratory illness (options A, E), ensuring adequate nutrition (option C), managing postural hypotension (option D) or addressing potential swallowing issues (option B). Given our patient's Parkinson's disease, along with increased lethargy and decreased ambulation that his daughter noticed, the presence of dysphagia is plausible. Dysphagia or difficulties in swallowing is common in Parkinson's disease due to impaired coordination of muscles in the mouth and throat. The reported change in tremors could also indicate an issue with the patient's medication timing, as escalating tremors might relate to "off" periods when the effect of Parkinson's medication begins to wear off before the next dose is due. This could potentially exacerbate dysphagia and further enhance aspiration risk. CONCLUDING: None of the other actions seem as immediately justifiable. While management of the pneumonia is ongoing, getting a CT scan (option A) or prescribing prophylactic levofloxacin (option E) treat the consequence and not the potential cause. Option C, placing a PEG tube, might be viable if severe dysphagia is confirmed, but it is invasive and thus not the first step. Fludrocortisone (option D) is used to manage orthostatic hypotension, a condition that our patient might have, but as his blood pressure is not critically low, it can be managed with non-pharmacological measures before prescribing medication. A swallowing evaluation (option B) in this context offers a more direct investigation into the potential dysphagia and aspiration risk, which matches the patient's clinical picture, and it can guide further specific interventions. RESPONSE: The most appropriate step before discharging this patient would be to perform a swallowing evaluation (B). This assessment would provide valuable information on whether the patient has any swallowing difficulties that could be increasing his risk of future pneumonia events due to aspiration. Additionally, it could provide a patient-specific plan to effectively manage these issues, hence improving his quality of life and reducing future hospital admissions.

**Facilitator** The consensus among the AI responses is that the most appropriate next step before discharging the 67-year-old Parkinson's patient, who was admitted for pneumonia treatment, is to obtain a swallowing evaluation (Option B). This decision is based on the patient's increased lethargy, decreased mobility, and occasional severe tremors, which are all symptoms of Parkinson's disease. These symptoms, coupled with the patient's recent pneumonia, suggest the possibility of dysphagia, a common issue in Parkinson's disease that can lead to aspiration and recurrent pneumonia. A swallowing evaluation would provide valuable information on whether the patient has any swallowing difficulties that could be increasing his risk of future pneumonia events due to aspiration. This assessment could also guide further specific interventions, improving his quality of life and reducing future hospital admissions. Other options, such as obtaining a CT scan of the chest, placing a PEG tube, prescribing fludrocortisone, or prescribing prophylactic levofloxacin, were considered but deemed less immediately justifiable given the patient's current clinical picture.
